# Complex Network Construction of Univariate Chaotic Time Series Based on Maximum Mean Discrepancy

**DOI:** 10.3390/e22020142

**Published:** 2020-01-24

**Authors:** Jiancheng Sun

**Affiliations:** School of Software and Internet of Things Engineering, Jiangxi University of Finance and Economics, Nanchang 330013, China; sunjc@jeufe.edu.cn; Tel.: +86-0791-8384-5702

**Keywords:** complex network, chaotic time series, Gaussian mixture model, maximum mean discrepancy

## Abstract

The analysis of chaotic time series is usually a challenging task due to its complexity. In this communication, a method of complex network construction is proposed for univariate chaotic time series, which provides a novel way to analyze time series. In the process of complex network construction, how to measure the similarity between the time series is a key problem to be solved. Due to the complexity of chaotic systems, the common metrics is hard to measure the similarity. Consequently, the proposed method first transforms univariate time series into high-dimensional phase space to increase its information, then uses Gaussian mixture model (GMM) to represent time series, and finally introduces maximum mean discrepancy (MMD) to measure the similarity between GMMs. The Lorenz system is used to validate the correctness and effectiveness of the proposed method for measuring the similarity.

## 1. Introduction

Chaotic time series exist widely in many fields, such as economics, physics, hydrology and so on [1]. In chaotic systems, the “butterfly effect” is a typical phenomenon in which small causes can have large effects [2]. Therefore, a chaotic system usually has highly complex behaviours, and the relevant analysis is a challenging task. 

In recent years, the application of complex network theory to time series analysis is increasing rapidly. Firstly, the time series was transformed into a network, and then, various complex network tools were used for analysis [3,4,5,6,7]. There are three kinds of network reconstruction methods: recurrence network based on phase space and visibility graphs and transition network based on Markov chain [6]. Regardless of the network construction method, a key problem to be solved is how to measure the similarity between nodes. For example, in order to measure the similarity between nodes, Euclidean distance, visual distance, and transition probability were applied to recurrence network [8], visibility graphs [9], and transition network [10], respectively. 

In this communication, we focus on the construction of a complex network of univariate chaotic time series, which is an effective way to analyse the time series [11]. Similarly, in this task, a core problem to be solved is how to measure the similarity between time series. In the community of time series analysis, some commonly used metrics, such as Euclidean distance [12], correlation coefficient [13], and dynamic time warping distance (DTW) [14], were used to measure similarities between time series. Especially for DTW, its outstanding advantage lies in the ability to find the optimal nonlinear alignment between two given sequences. However, most of the metrics cannot effectively measure the similarity in the case of chaotic time series. For example, time series from the same chaotic system are completely different in the form of local sub-sequences, which makes the pair matching metric, such as Euclidean distance, unable to measure the similarity between them. Even with statistical metrics such as the correlation coefficient, due to the rich structure of chaotic system, its effect is not as expected. In addition, the probability distribution of chaotic time series usually presents mixed distribution [15], which also brings challenges to measuring the similarity between sequences using statistical distances.

Considering the characteristics of chaotic time series mentioned above, in this communication, we improved the performance of similarity measurement from two aspects: (1) transform univariate time series (UTS) into a high-dimensional space to describe time series more accurately; (2) in the high-dimensional space, Gaussian mixture model (GMM) is used as the representation of time series, and distance metric is introduced to catch the similarity between GMMs.

## 2. Approach of Constructing Complex Networks 

In the complex network constructed, nodes represent the time series themselves, and the edges between nodes are determined by the strength of similarity between the time series. The process of constructing a network can be divided into the following sections.

### 2.1. Representation of Univariate Chaotic Time Series

We can realize the representation of UTS by using the idea of phase space reconstruction. Although a complex system is usually described by multiple variables, in most cases we can only observe a univariate (scalar) time series $T=\{x_{1},x_{2},\ldots ,x_{n}\}$ from the system, where n is the length of the time series. Fortunately, using the embedded theorem [16], we can reconstruct the original space of the system by unfolding the scalar time series into higher dimensional phase space. With the help of phase space reconstruction, we can investigate the geometric and dynamic properties of the original phase space as well as unobserved variables. In other words, it provides a new approach, which can transform UTS into state vectors in higher dimensional space, so as to describe and understand the characteristics of the system more accurately. This is exactly the motivation for representation of univariate time series.

By choosing an appropriate embedding dimension m and a time delay τ, we can transform a UTS $T=\{x_{1},x_{2},\ldots ,x_{n}\}$ into a state vector in the phase space as

(1) $$x_{i}={\left(x_{i},x_{i+\tau },x_{i+2\tau },\ldots ,x_{i+\left(m-1\right)\tau }\right)}^{T}$$

where m can also be regarded as the number of variables in the original phase space. Therefore, the phase space can be described by a m×(n−τ) matrix X, where each column represents the state point $x_{i}$ at time *i*, and each row represents a subsequence of the UTS. On the other hand, each row in X can also be viewed as observation of a variable. Consequently, X is multivariable time series (MTS) with m variables, which is converted from the UTS.

**Figure 1:**
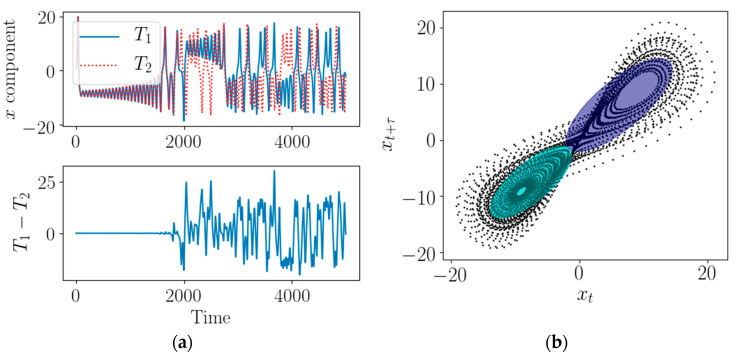
Chaotic time series of Lorenz system and its reconstructed phase space. (**a**) x component of Lorenz system. (**b**) Reconstructed phase space of $T_{1}$.

To illustrate the phase space reconstruction clearly, the well-known Lorenz system is used as an example to illustrate the reconstruction process [2]. The Lorenz system is described by three ordinary differential equations:

(2) $$\begin{array}{l} \frac{dx}{dt}=\sigma \left(y-x\right),\\ \frac{dy}{dt}=x\left(\rho -z\right)-y,\;\\ \frac{dz}{dt}=xy-\beta z \end{array}$$

where x, y, and z are system variables; *t* is the time, and σ, ρ, β are the system parameters. Two time series $T_{1}$ and $T_{2}$ (x component of the system) shown at the top of Figure 1a are generated using (2) with σ=8, ρ=28 and β=8/3. All system parameters are kept the same here, except that the initial conditions for generating $T_{1}$ and $T_{2}$ are slightly different by $10^{-2}$. The difference between the two-time series is shown at the bottom of Figure 1a. It can be seen from Figure 1a, in the beginning, the two-time series kept the same shape, but as time went on, the differences became larger and larger, and this phenomenon is known as the “butterfly effect”. In other words, although the two time series are generated from the same system with slightly different initial values, they are very different in local characteristics. Therefore, it is usually not feasible to measure the similarity between chaotic time series by general metric. In Figure 1b, pairs of time series values $x_{i}={\left(x_{i},x_{i+\tau }\right)}^{T}$ are plotted with black dots; this is the state vector in the reconstructed phase space of the x component of $T_{1}$ using (1). In other words, a system with two variables is reconstructed from the observation of one variable (a scalar time series). As can be seen from Figure 1b, more abundant geometric structure of the time series can be seen by using the phase space reconstruction, thus it can provide more information about the time series.

When the phase space is constructed, the next problem to be solved is how to represent the time series in the space. As mentioned above, the general point-to-point metrics are hard to measure the similarity between chaotic time series due to the complexity. Therefore, it is a more reasonable choice to calculate the statistical characteristics of time series and then measure the similarity between them. An intuitive way is to estimate the covariance matrix of the multivariable time series (MTS) in phase space and then calculate geodesic distance between them [17]. However, considering the complex structure of MTS in phase space, a single covariance matrix usually cannot accurately describe the data distribution of a chaotic system. For example, in Figure 1b, at least two independent Gaussian distribution models are required to accurately describe the Lorenz systems. Thus, a natural way here is to adopt multivariate GMM to represent MTS generated by chaotic systems. The GMM is given by

(3) $$G=\overset{n}{\underset{i=1}{\sum }}\alpha_{i}g_{i}=\overset{n}{\underset{i=1}{\sum }}\alpha_{i}N\left(\mu_{i},\Sigma_{i}\right)$$

where the *i*-th component is characterized by normal distributions $g_{i}=N\left(\mu_{i},\Sigma_{i}\right)$ with weights $\alpha_{i}$, means $\mu_{i}$, and covariance matrices $\Sigma_{i}$. In other words, GMM is a linear combination of several Gaussian distribution functions. In theory, GMM can fit any type of distribution, which is usually used to solve the case that one data set contains many different distributions. As shown in Figure 1b, GMM with two components is used to model MTS, and it (denoting as the ellipses) can well describe the double scroll structure of Lorenz system.

### 2.2. Complex Network Construction with Similarity Metric

Once the time series is represented by GMM, the similarity between time series is converted into the similarity between GMMs. Kullback–Leibler divergence is a commonly used solution to measure the distance between two probability distributions. However, it has no closed form solution in the case of GMM, and the implementation of Monte Carlo simulation becomes computationally expensive [18]. Therefore, we introduce maximum mean discrepancy (MMD) in reproducing kernel Hilbert spaces to quantify the similarity between GMMs [19]. Suppose we have two GMMs in ${\mathbb{R}}^{d}$:

(4) $$\begin{array}{c} P=\overset{m}{\underset{i=1}{\sum }}\alpha_{i}p_{i}=\overset{m}{\underset{i=1}{\sum }}\alpha_{i}N\left(\mu_{i},\Sigma_{i}\right)\\ Q=\overset{n}{\underset{j=1}{\sum }}\beta_{j}q_{j}=\overset{n}{\underset{j=1}{\sum }}\beta_{j}N\left(\mu_{j}^{\prime },\Sigma_{j}^{\prime }\right) \end{array}$$

where $p_{i}=N\left(\mu_{i},\Sigma_{i}\right)$; $q_{j}=N\left(\mu_{j}^{\prime },\Sigma_{j}^{\prime }\right)$; and m, n is the components number of P and Q, respectively. Given a kernel function $k\left(x,y\right)=\langle \varphi \left(x\right),\varphi {\left(y\right)\rangle }_{ℋ}$, the reproducing kernel Hilbert space (RKHS) ℋ corresponding to k(x,y) can be defined, where φ(x) is a feature mapping [20]. Given that we are in an RKHS, the mean map kernel can be defined as

(5) $$K\left(P,Q\right)=E_{x\sim P,y\sim Q}k\left(x,y\right)=\langle E_{x\sim P}\left[\varphi \left(x\right)\right],E_{y\sim Q}\left[\varphi \left(y\right)\right]\rangle$$

Then MMD can be easily defined as

(6) $$\begin{array}{cc} \mathrm{MMD}\left(P,Q\right) & =\|E_{x\sim P}\left[\varphi \left(x\right)\right]-E_{y\sim Q}\left[\varphi \left(y\right)\right]\|\\ & =\sqrt{K\left(P,P\right)+K\left(Q,Q\right)-2K\left(P,Q\right)} \end{array}$$

In the case of insufficient data, we can approximate the kernel function K(P,Q) by empirical estimation [21]:

(7) $$K_{\mathrm{emp}}\left(P,Q\right)=\frac{1}{n_{P}\cdot n_{Q}}\overset{n_{P}}{\underset{i=1}{\sum }}\overset{n_{Q}}{\underset{j=1}{\sum }}k\left(x_{i},y_{j}\right)$$

where ${\left\{x_{i}\right\}}_{i=1}^{n_{P}}$ and ${\left\{y_{j}\right\}}_{j=1}^{n_{Q}}$ are random samples. However, the approximation obtained with (7) introduces errors with high probability. Fortunately, when enough data is available, we can estimate the true distribution of the data; when GMM is used to approximate the distribution of the data, K(P,Q) has a closed solution:

(8) $$K\left(P,Q\right)=\sum_{i,j}\alpha_{i}\beta_{j}K\left(N\left(\mu_{i},\Sigma_{i}\right),N\left(\mu_{j}^{\prime },\Sigma_{j}^{\prime }\right)\right)$$

With (8), the form of K(P,P) and K(Q,Q) can be derived similarly. It turns out, introducing the Gaussian RBF kernel $k\left(x,y\right)=exp\left(-\gamma \|x-y{\|}^{2}/2\right)$, the product kernel of the Gaussian distribution is derived as:

(9) $$\left(N\left(\mu_{i},\Sigma_{i}\right),N\left(\mu_{j}^{\prime },\Sigma_{j}^{\prime }\right)\right)=1/{\left|\gamma \Sigma_{i}+\gamma \Sigma_{i}+I\right|}^{\frac{1}{2}}\exp\left(-\frac{1}{2}{\left(\mu_{i}-\mu_{j}^{\prime }\right)}^{T}{\left(\Sigma_{i}+\Sigma_{j}^{\prime }+\gamma^{-1}I\right)}^{-1}\left(\mu_{i}-\mu_{j}^{\prime }\right)\right)$$

With (6), (8), and (9), we can obtain the analytic form of MMD(P,Q) by introducing the Gaussian RBF kernel.

Once similarity measures are in place, the construction of complex networks is straightforward. First, each UTS is represented by a GMM, and then, MMD in (6) is used to calculate the distance between each pair of GMM to form a distance matrix $D=\left(\mathrm{MMD}\left(P_{i},Q_{j}\right)\right)$, where *i* and *j* denote different UTS. With a critical threshold $r_{c}$, D can be converted into adjacent matrix whose elements indicate whether pairs of nodes are connected or not in the network. An adjacent matrix $A=\left(a\left(P_{i},Q_{j}\right)\right)$, here $a\left(P_{i},Q_{j}\right)=1$ if $\mathrm{MMD}\left(P_{i},Q_{j}\right)\leq r_{c}$ and $a\left(P_{i},Q_{j}\right)=0$ if $\mathrm{MMD}\left(P_{i},Q_{j}\right)>r_{c}$. 

## 3. Experiments and Results

The Lorenz system in (2) has highly complex behaviors with the variation of the system parameters. With the change of system parameters, Lorenz system presents highly complex behavior. We randomly generate 150 time series of x components by keeping σ=10.0 and β=8/3 while varying ρ∈[28,45]. The reason is that (σ,ρ,β) form a vast three-dimensional parameter space. Considering the complexity of the Lorenz system, its characteristics have not been fully studied when σ and β take other values [22]. To simplify the problem, many researchers fix σ and β. while changing ρ. That is, each set of (σ,ρ,β) corresponds to a UTS and different parameter ρ corresponds to different class of time series. The length of each time series is 6000 data points, and the first 1500 points are removed to reduce the initialization effect of the system. The differential equation (2) is solved by scipy.integrate.odeint() in Python package SciPy [23], and the time point step is 0.01.

Firstly, with m=3, τ=8, each UTS is transformed into MTS in phase space by (1). Then, the GMM corresponding to each MTS is estimated, where the components number is 3. Finally, the MMD between the GMMs is calculated and eventually converted to the adjacency matrix. In addition, to evaluate the proposed method, three other metrics (geodesic distance, DTW and correlation coefficient) are also used to construct the network for comparison. By estimating the covariance matrix of MTS in phase space, the geodesic distance can be obtained and then a network formed [5]. For DTW and correlation coefficient, the metrics can be calculated directly between UTS.

**Figure 2:**
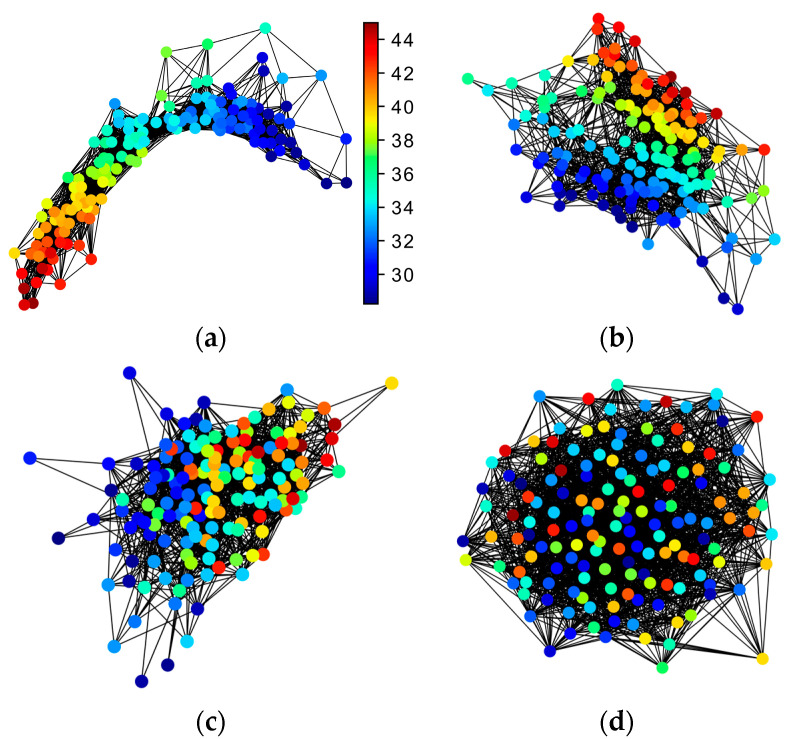
Construction of complex network based on different metric (colour bar denotes the value of ρ). (**a**) Network construction based on geodesic distance; (**b**) network construction based on maximum mean discrepancy (MMD); (**c**) network construction based on dynamic time warping distance (DTW); (**d**) network construction based on correlation coefficient.

The spring layout method in NetworkX package [24] was used to plot the network, and the results were shown in Figure 2. In the network, each node corresponds to a UTS, and the connection between nodes is determined by the adjacency matrix. The selected threshold $r_{c}$. enables 20% of the edges to be preserved to highlight the geometric structure of the network. The validity of network construction can be evaluated from two aspects: one is to see whether the similarity between nodes can be effectively captured; the other is to see whether the geometry of the network is conducive to the analysis of time series. In the first aspect, geodesic distance (Figure 2a) and MMD (Figure 2b) are better metrics of similarity because nodes with similar ρ are clustered together. In contrast, in Figure 2c,d, nodes with different ρ are mixed together, especially in Figure 2d, the nodes are completely confused and indistinguishable, like a random network, indicating that the metric used cannot effectively measure similarity. From the second aspect, the MMD is superior to the geodesic distance in the geometry of the network because the nodes in Figure 2a are squeezed together to make it difficult to distinguish. This phenomenon also indicates that MMD is more sensitive to measure similarity, which results in a looser network. In the following description, we will explain why a loose network structure is better than a tight one.

**Figure 3:**
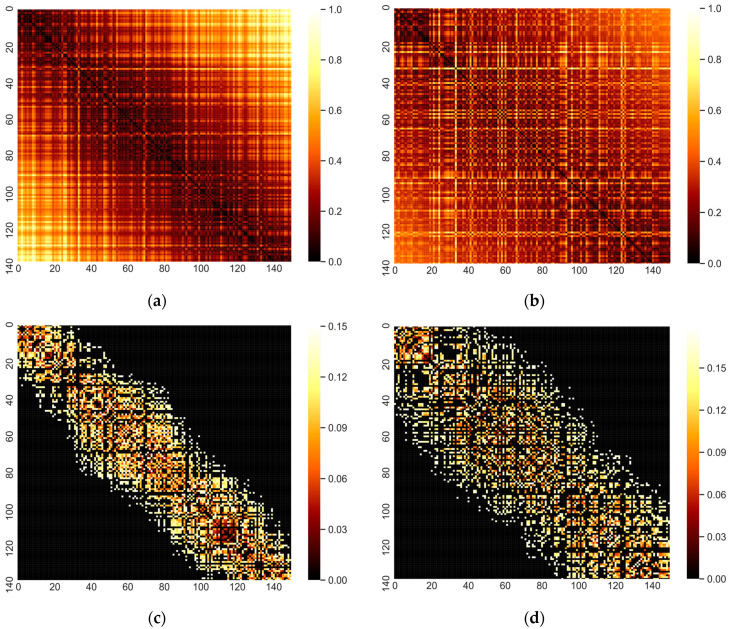
Heat map of distance matrix based on MMD and geodesic distance (coordinate label indicates the number of nodes and colour bar denote the value of distance between two nodes). (**a**) Heat map based on geodesic distance; (**b**) heat map based on MMD; (**c**) heat map in (**a**) with 20% of the edges to be preserved; (**d**) heat map in (**b**) with 20% of the edges to be preserved.

To analyse the characteristics of MMD and geodesic distance in detail, we show the heat map of the related distance matrix in Figure 3, which corresponds to the network structure. The size of the heat map is 150×150, corresponding to 150 nodes, and each pixel denotes the distance between a pair of nodes (time series). The nodes are arranged in ascending order according to the value of ρ. From Figure 3a,b, it can be seen that the distance near the diagonal is small (high similarity), otherwise the distance is large, which means that the node pairs with similar ρ have high similarity. To investigate this point more clearly, we set a certain threshold $r_{c}$ and retained the 20% of the edge (mentioned in Section 2.2), as shown in Figure 3c,d. As you can see, the reserved edges are centered diagonally and gradually spread to both sides. Therefore, if more edges are retained by increasing $r_{c}$, the topology of the network (the relationship between adjacent nodes) can still remain stable to some extent. 

By comparing Figure 3c,d (similar to Figure 2a,b), we can find that the network structure based on MMD is looser. From the characteristics of Lorenz system, the loose network structure is more reasonable. This is because small changes in ρ do not exactly correspond to smooth changes in the properties of time series. Although time series with similar ρ usually have similar properties, time series with different ρ sometimes have similar behaviors [25]. That is, a node should be similar to a node with a similar ρ, but it may also be similar to a node with a different ρ, which results in a loose network structure. Compared with geodesic method, MMD can capture the two similarities more effectively, and this results in a looser network. This is because the geodesic method is a special case of MMD in some ways. The deeper reason is that geodesic method uses only ONE covariance matrix (Gaussian distribution with a zero mean vector) to represent the data, while the MMD method uses GMM (linear combination of multiple Gaussian distributions) to fit the data. In contrast, MMD can capture more detailed information to find more neighbor nodes.

## 4. Conclusions

In this communication, a method was proposed for constructing a complex network of univariate chaotic time series. Compared with the commonly used metric, the introduced MMD can capture the similarity between GMMs more effectively, which is the key problem of constructing complex networks of the chaotic time series. Although the proposed method is specific to chaotic time series, it can also be applied to time series in other fields. In addition, it can be directly generalized to the case of multivariate time series by omitting phase space reconstruction.
